# Acute Myocardial Injury Assessed by High-Sensitivity Cardiac Troponin I Levels in Adult Patients with Early Sepsis at a Tertiary Referral Center in Mexico: An Exploratory Study

**DOI:** 10.3390/jcdd11010028

**Published:** 2024-01-18

**Authors:** Mauricio Alfredo Ambriz-Alarcón, Daniel Iván Arroyo-Espinosa, Héctor Meugniot-García, Juan Pablo Sánchez-Navarro, Brian Rafael Rubio-Mora, Sol Ramírez-Ochoa, Gabino Cervantes-Guevara, Miguel Robledo-Valdez, Alejandro González-Ojeda, Clotilde Fuentes-Orozco, Francisco Javier Hernández-Mora, Enrique Cervantes-Pérez

**Affiliations:** 1 Department of Internal Medicine, Centro Médico Nacional de Occidente “Lic. Ignacio García Téllez”, Instituto Mexicano del Seguro Social, Guadalajara 44350, Jalisco, Mexico; mau_ambriz@hotmail.com (M.A.A.-A.); arroyoivn@gmail.com (D.I.A.-E.); hecmg25@gmail.com (H.M.-G.); jp_sanchezn@hotmail.com (J.P.S.-N.); brianrrm@gmail.com (B.R.R.-M.); 2 Department of Internal Medicine, Hospital Civil de Guadalajara Fray Antonio Alcalde, Guadalajara 44350, Jalisco, Mexico; ramirez_ochoa_sol@hotmail.com; 3 Department of Gastroenterology, Hospital Civil de Guadalajara Fray Antonio Alcalde, Health Sciences University Center, Universidad de Guadalajara, Guadalajara 44350, Jalisco, Mexico; gabino_guevara@hotmail.com; 4 Department of Welfare and Sustainable Development, Centro Universitario del Norte, Universidad de Guadalajara, Colotlán 46200, Jalisco, Mexico; 5 Translational Nutrition Sciences Program, Health Sciences University Center, Universidad de Guadalajara, Guadalajara 44100, Jalisco, Mexico; miguel-antonio-97@hotmail.com; 6 Biomedical Research Unit 02, Hospital de Especialidades, Centro Médico Nacional de Occidente, Guadalajara 44350, Jalisco, Mexico; avygail5@gmail.com (A.G.-O.); clotilde.fuentes@gmail.com (C.F.-O.); 7 Department of Human Reproduction, Growth and Child Development, Health Sciences University Center, Universidad de Guadalajara, Guadalajara 44329, Jalisco, Mexico; frank.gine@gmail.com; 8 Department of Obstetrics, Hospital Civil de Guadalajara Fray Antonio Alcalde, Health Sciences University Center, Universidad de Guadalajara, Guadalajara 44350, Jalisco, Mexico; 9 Centro Universitario de Ciencias de la Salud, Universidad de Guadalajara, Guadalajara 44340, Jalisco, Mexico

**Keywords:** acute myocardial injury, high-sensitivity cardiac troponin I, sepsis

## Abstract

The objective of the study was to describe the frequency of acute myocardial injury (AMI) assessed by high-sensitivity cardiac troponin I (hs-cTnI) levels and to determine the possible initial risk factors (related to the characteristics of the patient, the disease, and the initial management) in a population of adult patients with early sepsis (within the first 72 h of diagnosis) in a single tertiary hospital center in western Mexico. For the inferential statistics, the proportions of the categorical dichotomous variables were compared using the chi-square test. In all analyses, *p* values less than 0.05 with a 95% confidence interval were considered significant. We included a total of 64 patients diagnosed with early sepsis, of whom 46 presented elevated hs-cTnI and were classified as having AMI. In our study, the frequency of AMI in patients with early sepsis was 71.87%, and no significant differences were found in all of the characteristics of patients with early sepsis with and without AMI, nor was any significant association found with any of the variables analyzed. In the population of western Mexico, the frequency of AMI in patients with early sepsis, assessed by hs-cTnI levels, is high and similar to that reported in other populations worldwide.

## 1. Introduction

Cardiac troponins I (cTnI) and T (cTnT) are components of the contractile apparatus of myocardial cells. An elevation in cTnI has not been reported to occur in response to damage to noncardiac tissues. However, this situation is more complex in the case of cTnT, where biochemical data indicate that injured skeletal muscles express proteins that the cTnT test can detect; so, in some clinical contexts, elevation of cTnT can come from skeletal muscle. cTnI and cTnT are the biomarkers of choice for the evaluation of myocardial injury, and the use of high-sensitivity cTn (hs-cTn) is recommended in routine clinical practice. The existence of myocardial injury is defined when blood cTn values are higher than the 99th percentile of the upper reference limit (URL) and may be acute (when there is a dynamic increase or a pattern of decrease in cTn values above the 99th percentile of URL in consecutive determinations) or chronic (when cTn values are persistently elevated) [1,2]. Nonischemic myocardial injury may occur as a consequence of various clinical settings affecting the heart, such as myocarditis, or may be related to noncardiac conditions, such as chronic kidney disease. Therefore, in patients with increased cTn values, it is necessary to differentiate between those who have suffered a nonischemic myocardial injury and those who have one of the subtypes of myocardial infarction. If there is no evidence of myocardial ischemia (ischemia denotes signs and/or symptoms of clinical myocardial ischemia), the diagnosis of myocardial injury must be made [2].

Patients should be classified as having acute or chronic myocardial injury based on a change in cTn concentration, ideally using trial-specific absolute delta criteria. In the absence of these criteria, those with cTn levels less than the 99th percentile at presentation with a greater than 50% increase in the 99th percentile URL on serial testing (and at least one value greater than the 99th percentile) are considered to have acute myocardial injury (AMI). On the other hand, when troponin concentrations are greater than the 99th percentile at the time of presentation, a relative change greater than 20% is compatible with AMI [3]. In patients who meet these criteria, a detailed clinical evaluation is required to determine the probability of coronary artery disease, since there are no risk estimation tools specific for these clinical settings; therefore, this evaluation is based on clinical judgment (analysis of presenting signs and symptoms, cardiovascular risk factors, and serial electrocardiographic findings) [3].

By definition, sepsis is a life-threatening organ dysfunction caused by a dysregulated host response to infection [4]. In 2017, there were an estimated 48.9 million incident cases of sepsis, with 11 million related deaths reported worldwide (19.7% of all global deaths) [5]. The heterogeneity of sepsis syndrome and septic shock makes it difficult to generate reproducible figures, with mortality rates ranging between 15 and 56%. Recently, a systematic review with meta-analysis found that the average mortality from septic shock at 30 days and 90 days was 34.7% and 38.5%, respectively; for sepsis, the average mortality at 30 days and 90 days was 24.4% and 32.2%, respectively (with variability between the geographic regions evaluated) [6].

One of the organs that can potentially be affected during the early process of sepsis is the heart muscle. The cardiac involvement associated with sepsis can manifest itself in multiple ways, either through an isolated primary acute myocardial cellular injury or also with a transient dysfunction that can range from imperceptible clinical implications to being potentially risky because it is related to a persistence of hemodynamic instability in established septic shock [2].

Elevations of hs-cTnI have been found in almost 70% of patients with sepsis during the first days of the illness [7]. Also, during episodes of bacteremia, an elevation in cTnI level can be detected in up to 43% of patients [8].

Sepsis-induced cardiomyopathy, also called “sepsis-induced myocardial dysfunction” (SIMD), is an increasingly recognized form of transient cardiac dysfunction in septic patients. While there are no formal definitions for SIMD, most review articles and expert opinions agree on some fundamental characteristics of this unique form of cardiac dysfunction: an acute and reversible alteration within the first 7–10 days of the disease, with global biventricular dysfunction (systolic and/or diastolic) and reduced contractility, left ventricular dilation, decreased response to fluid and vasopressor resuscitation, and absence of acute coronary syndrome [9]. Global hypokinesia of the left ventricle is very common in adult septic shock, observed in up to 60% of patients during the first three days of the disease [10].

Studies have shown that plasma concentrations of hs-cTn, as a biomarker of myocardial injury, even correlate well with the functional abnormalities observed in echocardiography of patients with sepsis and septic shock in the first days of diagnosis, especially with diastolic dysfunction of the left ventricular and right ventricular dilation [11] and left ventricular systolic dysfunction [12]. In patients with septic shock, up to 65% of those with an elevated hs-cTnI level have various cardiac dysfunctions on transthoracic echocardiography and, more than the baseline level, a maximum change in hs-cTnI and ST waves-T on the electrocardiogram can be considered a risk factor for the development of SIMD [13].

A descriptive study from Mexico included 68 emergency medical services, where 2379 patients were treated. The prevalence of sepsis was 12.9% with an overall mortality of 16.93%, which in cases of sepsis was 9.39% and in cases of septic shock was 65.85% (the latter with a mortality higher than that reported internationally) [14]. Also, in a recent study of the Mexican population, it was found that of 65 patients analyzed who presented with septic shock and who were admitted to intensive care, 46% had a high serum cTnI in the first 24 h, with his being a poor predictor of mortality in these patients [15]. However, a recent systematic review with meta-analysis (including twenty-four prospective studies and four retrospective studies) demonstrated that elevated cardiac troponin levels in patients with sepsis were a predictor of in-hospital and long-term mortality [16].

In a recent study [17] conducted in patients with sepsis admitted to the intensive care unit (ICU), highly sensitive measurements of cardiac troponin I, as well as other plasma biomarkers related to inflammation, coagulation, and endothelial function, were taken. For that study, 88% of the 1037 eligible patients were analyzed, and 61% had elevated troponin I levels at admission. After adjustment for age and cardiovascular comorbidities, the analysis showed that activated coagulation was independently associated with troponin elevation during sepsis (standardized regression coefficient: 0.55; 95% confidence interval, CI: 0.257–0.845, *p* < 0.001), while hyperinflammation and endothelial dysfunction were not. These findings suggest that myocardial injury (MI) during sepsis is mediated by the systemic activation of coagulation rather than by circulating inflammatory mediators or endothelial activation [17].

The main objective of this study was to describe the frequency of acute myocardial injury (AMI) in patients with early sepsis (within the first 72 h) in a tertiary hospital in western Mexico, using the measurement of hs-cTnI (and from this, establish the level of elevation above the URL). Among the secondary objectives was to determine the probable initial risk factors (related to the characteristics of the patient, the disease, and the initial management) for the development of acute myocardial injury in this clinical context.

## 2. Materials and Methods

For this retrospective study, data were collected from patients diagnosed with sepsis who were hospitalized and/or hospitalized adult patients who developed sepsis who were evaluated by the Internal Medicine service at the Hospital de Especialidades del Centro Médico Nacional de Occidente (CMNO) during the period from March 2019 to March 2022. Routinely, both in the emergency department and during hospitalization, those patients with initial suspicion of sepsis and who required resuscitation due to hypotension had an initial level of hs-cTnI taken, and if it was above the URL, subsequent determinations of hs-cTnI were taken for monitoring.

Inclusion criteria: patients of either sex; age from 18 to 75 years; confirmed sepsis (defined according to the criteria of the 2016 Surviving Sepsis Campaign [4]) of pulmonary, abdominal, urinary, soft tissue, bone, central nervous system, or bloodstream infectious origin (related to intravascular devices); baseline hs-cTnI measurement at least in the first 72 h from sepsis diagnosis.

Exclusion criteria: suspicion or confirmation of acute coronary syndrome (with typical or atypical symptoms) concomitant with the sepsis process; cardiopulmonary resuscitation during hospitalization; diagnosis or suspicion of myocarditis, pericarditis, or endocarditis; recent cardiac surgery (less than two weeks); confirmed diagnosis of COVID-19.

For the measurement of high-sensitivity troponin I levels, the VIDAS^®^ standardized ultrasensitive assay (bioMériux, Marcy-l’Étoile, France) was used, which is an automated quantitative test that allows for the presence of human cardiac troponin I in human serum or plasma to be determined using the ELFA technique (Enzyme Linked Fluorescence Assay). The 99th percentile value for the overall population is 19 ng/L (CI: 15–38); for men 25 ng/L (CI: 17–50) and for women 11 ng/L (CI: 8–29). For the purposes of this study, MI was defined as a high-sensitivity cardiac troponin I (hs-cTnI) level above 19 ng/L in at least one measurement within the first 72 h after the diagnosis of sepsis.

Detailed information on the characteristics, comorbidities, and disease status of each of the included patients was collected retrospectively and cross-sectionally using data from the clinical record and entered into a specific data collection sheet in Excel 2021 (Microsoft, Albuquerque, NM, USA).

For the statistical analysis, the GraphPad Prism program (version 9.3.1.471, GraphPad by Dotmatics, Bishop’s Stortford, UK) was used.

For the descriptive statistics of the qualitative variables, both the absolute frequencies and the relative frequencies were calculated, and the data were presented as percentages and proportions. For the inferential statistics, the proportions of the categorical dichotomous variables were placed in a 2 × 2 contingency table to compare them using the chi-square test.

In all analyses, *p* values less than 0.05 with a 95% confidence interval were considered significant.

To calculate the frequency of AMI in adult patients with early sepsis, the following equation was used:

$$\mathrm{Frequency}=\frac{\mathrm{Total}\;\mathrm{number}\;\mathrm{of}\;\mathrm{individuals}\;\mathrm{with}\;\mathrm{elevated}\;\mathrm{hs}\;\mathrm{cTnI}\;\mathrm{levels}}{\mathrm{Total}\;\mathrm{number}\;\mathrm{of}\;\mathrm{individuals}\;\mathrm{with}\;\mathrm{early}\;\mathrm{sepsis}\;\mathrm{who}\;\mathrm{met}\;\mathrm{the}\;\mathrm{elegibility}\;\mathrm{criteria}}$$

The study was conducted in accordance with the Declaration of Helsinki. The Clinical Research and Bioethics Committee of the Centro Medico Nacional de Occidente approved this study (R-2022-1301-155), and the requirement for informed consent for this retrospective analysis was waived.

## 3. Results

During the study period, a total of 95 patients who were diagnosed with sepsis and had plasma hs-cTnI concentration measurements were identified. Of these, 64 patients met the inclusion criteria. Thirty-one patients were excluded from the study because they did not meet the established deadline for hs-cTnI measurement (which had to be taken in the first 72 h) and/or met any exclusion criteria.

In our study, the frequency of AMI identified by an elevated hs-cTnI level in hospitalized patients with early sepsis was 71.87%, with a median level of 191 ng/L (interquartile range: 21.5–212.5).

The range of troponin elevation was classified as low (20–100 ng/L), moderate (101–500 ng/L), and high (>500 ng/L) [7], as presented in Table 1.

Table 2 presents the comparison of patient-related variables (sex, advanced age, smoking, defined as at least 10 cigarettes per day within 10 years prior to study inclusion, obesity, diabetes mellitus, systemic arterial hypertension, chronic kidney disease, previous myocardial infarction, and heart failure) with the disease (site of infection, acute kidney injury, recent surgery, and the Sequential Organ Failure Assessment (SOFA) score, calculated in the first 24 h of diagnosis of sepsis; a cutoff of 10 points or higher was used, which confers a mortality risk of 50% or more) and in relation to the initial treatment (invasive mechanical ventilation and septic shock requiring vasopressor, both starting within the first 24 h of sepsis diagnosis). The mortality rate of the patients included in our study was 50% (n = 32). Of the patients without elevated hs-cTnI, 33.33% (n = 6) died, while 56.52% (n = 26) of the patients with elevated hs-cTnI died. However, this difference was not statistically significant (OR 0.38, 95% CI: 0.11–1.16, *p* = 0.0953).

## 4. Discussion

In this population study from western Mexico, we found that the frequency of AMI in patients with early sepsis, evaluated using an ultrasensitive method such as the detection of hs-cTnI levels, was 71.87%. It should be noted that, with respect to the variables included, a considerably lower proportion of patients with AMI presented with obesity and systemic arterial hypertension, but those with an SOFA score ≥10 points and those who required management with invasive mechanical ventilation in the first 24 h presented a higher proportion of AMI (without finding a statistically significant association). A study with a larger sample size could confirm whether our findings are significant or coincidental. In a prospective study conducted by Frencken et al. [7], patients with sepsis were identified in two Dutch intensive care units between 2011 and 2013, measuring the plasma concentration of hs-cTnI daily during the first four days of stay. Of 1258 patients with sepsis, 1124 (89%) were eligible for the study, finding that the hs-cTnI concentration was elevated in 60% of patients on day one, and 67% continually presented elevated hs-cTnI levels. For the cTnI during the first four days, the median plasma hs-cTnI concentrations on days 1 to 4 were 109 ng/L (IQR, 39–394), 103 ng/L (IQR, 38–449), 79 ng/L (IQR, 31–281), and 82 ng/L (IQR, 32–253), respectively, compared to the median found in our study, which was 191 ng/L (IQR, 21.5–212.5). Although the frequency observed in our study is similar to that reported in the study by Frencken et al. [7], it is important to note that the latter reported prevalence, whereas our result only indicates that a significant number of sepsis patients appear to have AMI, even in a small sample. Failure to consider this pathology could result in delayed detection. Based on the current international literature [17], this finding may have clinical significance. However, further studies are needed to confirm its significance.

In our study, a low hs-cTnI elevation range was found (20–100 ng/L) in 39.13% of patients, a moderate (101–500 ng/L) range in 36.95% of patients, and a high (>500 ng/L) range in 23.91% of patients. This stratification was decided because, in the study carried out by Frencken et al. [7], Cox regression analysis revealed that elevated hs-cTnI concentrations were associated with higher mortality rates during the first 14 days, with an adjusted hazard ratio: 1.72 (95% CI; 1.14–2.59) and risk ratio: 1.70 (95% CI; 1.10–2.62) for concentrations of 100–500 ng/L and >500 ng/L, respectively, but not at a later time. However, in a retrospective cohort study of patients older than 40 years who survived to hospital discharge after an index hospitalization for sepsis, elevation of cTnI during sepsis identified patients at increased risk for subsequent cardiovascular complications; among 14,046 eligible adults with measured cTnI, 2012 (14.3%) experienced the composite cardiovascular outcome, including 832 (10.9%) patients with normal cTnI levels, compared with 370 (17.3%), 376 (17.6%), and 434 (20.3%) patients within each tertile of sequential abnormal cTnI, respectively (*p* < 0.001). Patients within high cTnI tertiles had higher risks of adverse cardiovascular events, with a cTnI-adjusted hazard ratio of 0.04–0.09 ng/mL: 1.37 (95% CI: 1.20–1.55); adjusted hazard ratio for cTnI 0.09–0.42 ng/mL: 1.44 (95% CI: 1.27–1.63); and adjusted hazard ratio for cTnI > 0.42 ng/mL: 1.77 (95% CI: 1.56–2.00) [18]. Due to the small sample size in our study, we did not find significant differences with the evaluated risk factors. Therefore, evaluating the relationship between the elevation levels of hs-cTnI and these risk factors would provide data that are difficult to interpret since the sample size of patients would be even smaller and not representative of the population. In future studies with a larger sample size, it is important to investigate the possibility that higher levels of hs-cTnI, and therefore higher AMI, may result in greater mortality and associated risks [18].

During hospitalization, 50% (n = 32) of the patients in our study died. Of these, six patients (33.33%) were in the group without elevation of hs-cTnI and twenty-six patients (56.52%) were in the group with elevation of hs-cTnI. Although a greater proportion of patients with elevated hs-cTnI died during hospitalization, the comparison between groups was not significant (*p* = 0.953). This lack of significance may be due to the small size of our patient sample. The interpretation of these results should be approached with caution as the observed mortality may not be causally related to the elevation of hs-cTnI. Additionally, information on the short- and long-term mortality of surviving patients after hospitalization was not available. Therefore, subsequent prospective studies with a more balanced selection of patients are needed to better understand the effect of AMI on the mortality of patients with sepsis.

Even though our study showed that patients with high concentrations of hs-cTnI had higher rates of smoking, diabetes mellitus, chronic kidney disease, previous myocardial infarction, heart failure, recent surgery, elevated SOFA scores (≥10), invasive mechanical ventilation, and shock with vasopressor requirement, none of these variables were statistically significant, and the associations of risk factors with a small sample size, which may or may not be representative of the population, are difficult to interpret. However, future studies could confirm or rule out the significance of these initial findings. The study by Vasile et al. [19], where diabetes mellitus, previous myocardial infarction, heart failure, chronic dialysis, and an elevated APACHE III score occurred in a higher proportion among patients who presented elevated cardiac troponin T levels at admission, with most of the results being statistically significant, is an example of such research. Additionally, in the study by Frencken et al. [7], patients with elevated hs-cTnI levels showed a higher proportion of diabetes mellitus, chronic kidney disease, heart failure, previous myocardial infarction, and vasopressor requirement, all with statistically significant differences.

Our study is one of the first conducted in the Mexican population that used an ultrasensitive biomarker (highly sensitive and specific for cardiac muscle) for the detection of AMI in patients with early sepsis. However, it has several important limitations. The main limitation lies in the retrospective design and the fact that the period established for data capture covered the beginning of the COVID-19 pandemic and the highest peak of cases related to the pandemic, which produced a lower number of patients admitted for sepsis not related to COVID-19. However, the socioeconomic conditions of the public health system in Mexico mean that access to this test is limited in our country, which is why this descriptive study is important, despite the small number of patients and its retrospective design. The findings of our study, compared to those reported in previous studies, should be interpreted with caution, since it is likely that the frequency in our study is higher than the real one due to the sample size that was analyzed. However, it is useful as a sample of the frequency with which it occurs since it is usually not detected or considered in many hospital settings in our country. Furthermore, given that the total number of patients included was small, it was expected that the association results would not be significant, despite the conditions and pathologies that have been associated with the development of acute myocardial injury in patients with sepsis in previous studies. Last, in future studies, in addition to using a prospective design, which would facilitate subsequent follow-up for each patient, it would be interesting to be able to make associations with short- and long-term mortality with the different categories of hs-cTnI elevation proposed here and in the literature [7], in the Mexican population. Due to limitations in accessing patient clinical information, such as the lack of digitization of clinical records in some departments of our hospital, the mortality data and the context of the population from which our patient samples were taken are very limited. Therefore, our results should be interpreted based on the exploratory nature of our study.

Sepsis-induced myocardial dysfunction is recognized as a major contributor to the persistent hemodynamic instability in patients with septic shock [20], with a potentially deleterious contributing effect on the clinical outcome and hospital mortality.

## 5. Conclusions

In the population of western Mexico, the frequency of AMI in patients with early sepsis, assessed by high-sensitivity cardiac troponin I levels, is high and possibly similar to that reported in other populations worldwide. However, a study with a prospective design and a larger sample size could provide a more precise idea of the magnitude of the problem and possibly confirm the variables associated with AMI in the Mexican population, if any.

## Figures and Tables

**Table 1:** Elevation range of high-sensitivity troponin I levels in patients with myocardial injury.

TnI Elevation Range	Patients with MI, *n* = 46 (100%)
Low (20–100 ng/L)	18 (39.13%)
Moderate (101–500 ng/L)	17 (36.95%)
High (>500 ng/L)	11 (23.91%)

TnI = troponin I; MI = myocardial injury.

**Table 2:** Comparison of patient characteristics between patients without acute myocardial injury and those with acute myocardial injury.

Variable	Without AMI n = 18 (28.13%)	With AMI n = 46 (71.87%)	Odds Ratio (CI 95%)	*p*
Demographics characteristics				
Age > 60 (years), n (%)	13 (72.22)	27 (58.70)	1.83 (0.57–5.38)	0.3149
Male sex, n (%)	11 (61.11)	26 (56.52)	0.82 (0.28–2.47)	0.7382
Medical history				
Smoking, n (%)	3 (16.67)	11 (23.91)	0.63 (0.17–2.33)	0.5284
Obesity, n (%)	6 (33.33)	7 (15.22)	2.78 (0.80–10.26)	0.1053
Diabetes mellitus, n (%)	8 (44.44)	26 (56.52)	0.61 (0.21–1.84)	0.3840
HBP, n (%)	15 (83.33)	27 (58.70)	3.5 (0.90–12.50)	0.0621
Chronic kidney disease, n (%)	3 (16.67)	10 (21.74)	0.72 (0.19–2.73)	0.6502
Previous acute myocardial infarction, n (%)	2 (11.11)	7 (15.22)	0.69 (0.13–3.10)	0.6709
Heart failure, n (%)	4 (22.22)	21 (23.91)	0.90 (0.28–3)	0.8858
Disease characteristics				
Infection site:				
Lung, n (%)	0 (0)	12 (26.09)	-	-
Abdominal, n (%)	5 (27.78)	10 (21.74)	1.38 (0.42–4.34)	0.6081
Urinary tract, n (%)	9 (50)	16 (34.78)	1.87 (0.59–6.01)	0.2619
Soft tissue, n (%)	3 (16.67)	7 (15.22)	1.11 (0.28–5.18)	0.8858
Central nervous system, n (%)	0 (0)	0 (0)	-	-
Bloodstream (by devices), n (%)	1 (5.56)	1 (2.17)	2.64 (0.13–51.22)	0.4845
Acute kidney injury, n (%)	12 (66.67)	30 (65.22)	1.06 (0.33–3.64)	0.9126
Recent surgery, n (%)	4 (22.22)	11 (23.91)	0.90 (0.28–3)	0.8858
Elevated SOFA score (≥10), n (%)	2 (11.11)	13 (28.26)	0.31 (0.06–1.37)	0.1453
Treatment characteristics				
Invasive mechanical ventilation, n (%)	4 (22.22)	18 (39.13)	0.44 (0.14–1.53)	0.2004
Shock with vasopressor requirement, n (%)	11 (61.11)	33 (71.74)	0.61 (0.18–1.87)	0.4095

CI = confidence interval; AMI = acute myocardial injury; SOFA = Sequential Organ Failure Assessment; HBP = High Blood Pressure.

## Data Availability

The data presented in this study are available on request from the corresponding author. The data are not publicly available due to privacy reasons.
